# A Systematic Review Evaluating the Impact of Glycemic Control on Mortality and Major Adverse Cardiac Events in Type 2 Diabetic Patients With Acute Coronary Syndrome

**DOI:** 10.7759/cureus.109179

**Published:** 2026-05-19

**Authors:** Krishna Adhikari, Asad Ullah, Xu Bing

**Affiliations:** 1 Cardiology, Northern Jiangsu People's Hospital Affiliated to Yangzhou University, Yangzhou, CHN

**Keywords:** acute coronary syndrome, diabetes mellitus type 2, glycemic control, hyperglycemia, major adverse cardiac event (mace)

## Abstract

Acute coronary syndrome (ACS) is a global cause of mortality and morbidity, specifically among type 2 diabetes mellitus (T2DM) patients. Patients with hyperglycemia had unfavorable outcomes after acute myocardial infarction (AMI), with a high rate of mortality ranging between 1.5 and 2.5 fold, along with a high incidence of major adverse cardiovascular events (MACEs), compared to normoglycemic patients. Acute hyperglycemia and chronic glycemic exposure are indicated by HbA1c and are related to poor cardiovascular outcomes, which were followed by ACS. The review assessed the current evidence on the effect of the glycemic status and the strategies to control the glycemic level among T2DM patients presenting with ACS. This was a systematic review that was conducted according to the PRISMA guidelines. PubMed/MEDLINE, Web of Science, and Scopus were used as search engines to get relevant studies for the review. Eligible studies included those with ACS patients, and the glycemic status was investigated in association with the clinical outcome, such as mortality and MACEs. The Newcastle-Ottawa Scale was used to assess the quality of the study. Twelve studies included 85,000 patients; the admission of hyperglycemia was strongly related to the worst outcomes after AMI. The all-cause mortality ranges from 9.0% to 25.1%, while the variation in hospital mortality ranges between 1.9% and 9.8%. Various studies have included the high risk associated with high glucose levels. Hyperglycemia was associated with a high rate of adverse cardiac events, including heart failure, cardiogenic shock, and arrhythmias. The study concluded that poor glycemic control increases the rate of mortality during admission, and the adverse cardiac events are also enhanced among type 2 diabetes patients with ACS.

## Introduction and background

Diabetes is associated with cardiovascular (CV) disease, specifically coronary artery disease (CAD). Chronic hyperglycemia is associated with a high risk of adverse complications related to CV disease; thus, a low level of glycemia can improve the clinical outcome among diabetes patients. However, controversies exist about the management of glucose among diabetes patients with CAD. Several clinical trials showed that strict glucose control may lead to unfavorable clinical outcomes along with a high risk of severe hypoglycemia [1]. Thus, it is important to detect the specific population that would benefit from managing glucose. Resistance to insulin has been regarded as the most significant determinant of CV risk for predicting future CV incidents [2]. The triglyceride-glucose (TyG) index, which is calculated from fasting triglycerides (TGs) and fasting blood glucose levels, has recently been considered a simple and most reliable indicator of insulin resistance. The formula for calculation is TyG index = ln [fasting triglycerides (mg/dL) × fasting blood glucose (mg/dL) / 2]. The TyG index has been identified as a biomarker for predicting the prevalence and CAD prognosis in cohorts of CAD primary and secondary prevention populations [3]. Substantial advances have been made in the management of acute myocardial infarction (AMI), with early revascularization now established as the standard of care. Nevertheless, individuals with diabetes who experience AMI continue to represent a high-risk subgroup, exhibiting a persistently elevated incidence of complications and adverse clinical outcomes. These consist of death, heart failure, stroke, and nonfatal reinfarction, whilst an in-patient, within one month, 6 to 12 months, or prolonged duration [4]. Individuals with polyvascular disease, characterized by atherosclerotic involvement of two or more vascular beds, including the coronary, cerebral, or peripheral arteries, are at significantly elevated CV risk. Patients who have experienced acute myocardial infarction (AMI) and subsequently develop clinical manifestations of heart failure or left ventricular dysfunction are particularly vulnerable to adverse outcomes. The coexistence of comorbid conditions such as diabetes mellitus further amplifies the risk of complications and contributes substantially to increased mortality [5]. Dysglycemia can significantly cause oxidative stress, with endothelial and platelet impairment and the upregulation of inflammation. These are strongly associated with unfavorable outcomes. Recently, there has been no consensus regarding the benefits of glycemic control apart from the acute peri-infarct period and other evidence to support the patient. New studies reported the epigenetic alterations following acute and sustained hyperglycemia behind the idea of vascular hyperglycemic memory, where the end-organ failure from the irregularity of glucose levels is irreversible [6]. The study aims to assess the effect of glycemic control on the rate of mortality and the major adverse cardiac conditions among type 2 diabetes mellitus (T2DM) patients with acute coronary syndrome (ACS). The study seeks to evaluate the relationship between hyperglycemia, treatment modalities and clinical outcomes to identify the optimum glycemic target to improve the prognosis among high-risk populations [5,6].

## Review

Methods

Study Design and Reporting Standards

The study is a systematic review to evaluate the effects of glycemic control on the rate of mortality and major cardiac events among T2DM patients with ACS. The study was conducted for a period of four months from November 2025 to February 2026. The review was conducted and reported on the basis of the Preferred Reporting Items of Systematic Reviews and Meta-Analyses (PRISMA) guidelines to ensure future modifications of the study, to maintain the reproducibility and transparency of the study.

Database Searching and Screening

An extensive and systematic literature review was performed to assess the relationship between the glycemic status or glycemic control strategies and the clinical outcomes among patients with T2DM who had ACS. The electronic databases which were used for search include the PubMed/MEDLINE, Web of Science, Scopus, Embase, Science Direct and DOAJ, and the search was done to most recently available date by the use of predefined search strategy, in combination of terms like “diabetes” OR “diabetic” AND “acute myocardial infarction” OR “AMI” AND “hyperglycaemia” OR “glucose level.” All duplicate records were detected and removed before the screening process. The selection of the study was performed in two stages. Four independent reviewers screened the titles and abstracts according to the predefined inclusion criteria, which was followed by the full-text evaluation of the eligible studies. The selection of the study was conducted in two stages. Four independent reviewers screened the titles and the abstracts for the detection of the relevant studies on the basis of the predefined criteria, which was followed by the detailed full-text assessment for enrollment. The disagreements of the selection process were resolved by discussion, while the final decisions were made by the senior author. The structured framework of the search strategy and eligibility criteria was developed on the basis of the PICO. The population of patients included those with T2DM who presented with ACS, with the intervention/exposure consisting of the glycemic status or control measures like admission hyperglycemia, HbA1c levels, intensive or protocol-based glucose control, insulin therapy, and other glucose-lowering treatments. The comparison consists of the standard or suboptimal glycemic control, with low HbA1c levels or normoglycemia. The outcome of the interest consists of the major adverse cardiac events (MACEs) and all-cause mortality, including CV death, stroke, and myocardial infarction.

Inclusion and Exclusion Criteria

Inclusion criteria: Studies that include the diagnosis of patients with AMI or ACS and those including the ST-elevation myocardial infarction, non-ST-elevation myocardial infarction, or unstable angina were included. Studies discussing patients with both diabetes and without diabetes were included, and studies where the glycemic parameters, such as admission hyperglycemia, fasting or random blood glucose, stress hyperglycemia ratio, and HbA1c levels, were evaluated were included in the study. Studies that involved patients who were affected by HbA1c reliability, such as anemia or sickle cell disease, were excluded from the study. Studies that compared high glucose levels with normal or low glycemic levels were included in the study. Studies that reported the glycemic status, specifically the mortality, CV mortality, in-hospital mortality, short-term mortality, or long-term mortality, were included. Full-text English articles, RCTs, cohort studies, case-control studies, registry-based studies, or some cross-sectional observational studies were included.

Exclusion criteria: Studies involving populations other than AMI or ACS were excluded. Studies that did not evaluate glycemic status, hyperglycemia, or glucose-related parameters as an exposure variable were also excluded. Those that are irrelevant and not related to mortality or duplicate studies were not considered. The included studies involved the type of inpatient pharmacologic therapy that was used for hyperglycemia management, as the crucial objective was to assess the relationship between the glycemic status and clinical outcomes in comparison to treatment effects. Studies that were case reports, case series, narrative reviews, systematic reviews, editorials, or conferences that lacked the original data were not considered. Studies for which the full text was unavailable were excluded.

Quality Assessment

The methodology of the observational cohort studies was evaluated by the use of the Newcastle-Ottawa Scale, which was recommended by the Cochrane Collaboration. This tool assesses the quality of the study across three domains, including the selection of the study groups, the comparison between the cohorts, and outcome assessment. The scale provides the highest score as 9 stars, each star per item, except the comparability domain, which allows for a maximum of 2 stars. On the basis of the total score, studies were categorized as poor quality (0-3 stars), moderate quality (4-6 stars), or high quality (7-9 stars). The quality was evaluated on the basis of the reviewers, and disagreements were resolved by discussion and consensus.

Data Extraction

Extraction of data was independently performed by two reviewers by the use of a predesigned and piloted data extraction form to maintain consistency and reproducibility. Discrepancies were resolved by discussion, while adjudication was performed by the third reviewer. Both reviewers performed data extraction by the use of predesigned and piloted data extraction form to maintain consistency and reproducibility. The third reviewer evaluated the studies and made the final decision on the basis of the exclusion and inclusion criteria. The extracted data consist of the author, year, design of the study, country, recruitment time, size of sample, population features, and the comparative groups. Particularly, focus was given to the definitions associated with glycemic status, like the time and the timing of glucose, the used indices, and the classification of the type and status of diabetes. The outcome variables were collected, such as the in-hospital, short-term, and long-term mortality, CV outcomes, and adverse events like major adverse cardiac events. Statistical methods, effect measures, and covariates include the multivariable analyses, follow-up duration and the bias assessment by the use of the NOS to ensure qualitative synthesis.

Results

**Figure 1:**
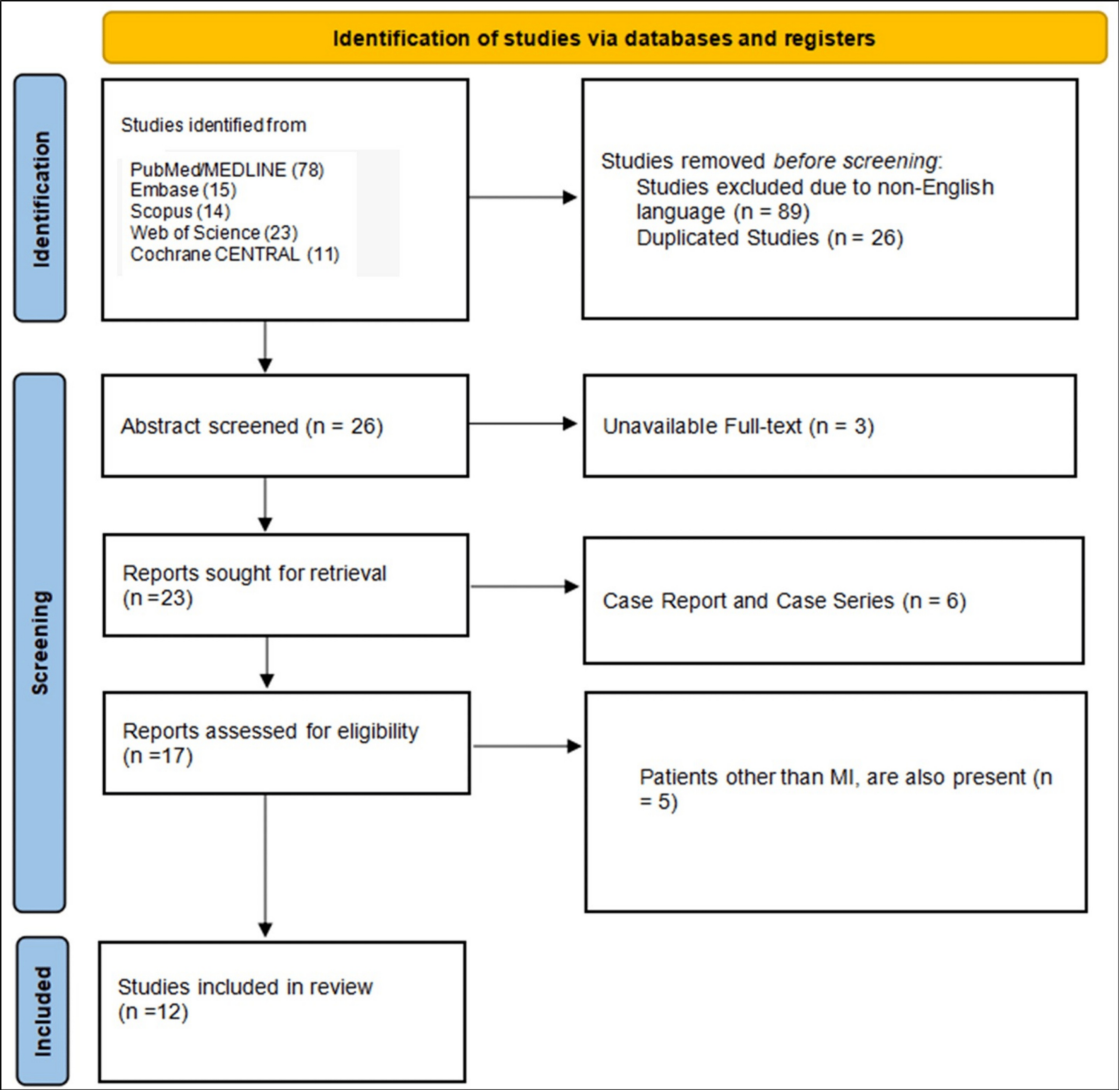
PRISMA flowchart PRISMA: Preferred Reporting Items of Systematic Reviews and Meta-Analyses

The PRISMA flowchart demonstrated the process utilized to identify, screen, and assess the final patient selection. A total of 141 studies were detected from the electronic databases, including 78 studies from PubMed/MEDLINE, 15 studies from Embase, 14 studies from Scopus, 23 studies from Web of Science, and 11 studies from the Cochrane CENTRAL database. Some of the studies were removed during the screening process. Eighty-nine studies were excluded and were published in non-English languages. Twenty-six extra records were removed as duplicate records. The remaining 26 studies were used for abstract screening. Out of them, three articles were excluded because of the unavailability of the full text, and only 23 reports were sought for full-text retrieval. Furthermore, six studies were categorized as case reports or case series. As a result, only 17 studies were evaluated for eligibility, which led to the exclusion of five studies as patients with conditions other than myocardial infarction were included. After the screening of patients and the pre-defined criteria, a total of 12 studies were included and incorporated into the review (Figure 1).

Table 1 demonstrates 12 observational studies that evaluated the association between the glycemic status and outcomes among patients with AMI. Most of the studies were retrospective studies or cohort or registry bases study, indicating the real-world clinical data. The size of samples varies within the range from 277 patients to 45,468 patients. The combined population increased by 80,000 participants, which indicated a strong statistical analysis. The investigation had compared the groups on the basis of the glycemic status, such as hyperglycemia versus normoglycemia or the diabetic versus non-diabetic AMI patients. Some literature studies utilized the admission of glucose or categorized patients on the basis of glucose regulation, as observed in the study by Cui et al. (2022) [7]. The table showed balanced group distributions (Table 1).

**Table 1:** Characteristics of the included studies AMI: Acute myocardial infarction; MIOCA: myocardial infarction with obstructive coronary arteries; MINOCA: myocardial infarction with non-obstructive coronary arteries; ABG: admission blood glucose

Author	Design	Country	Year	Group 1	Group 2	Total patients	Group 1 (n)	Group 2 (n)
Cui et al., (2022) [7]	Prospective, nationwide, multicenter observational registry	China	2013–2014	Low ABG (below subgroup-specific cutoff)	High ABG (≥ subgroup-specific cutoff)	6,892	DM: 1,139 Pre-DM: 1,263 NGR: 1,055	DM: 1,681 Pre-DM: 748 NGR: 1,006
Ritsinger et al., (2019) [8]	Registry-based randomized cohort analysis	Sweden	2019	Elevated admission plasma glucose AMI patients	Normal admission plasma glucose AMI patients	5,309	2,779	2,530
Yuan et al., (2023) [9]	Retrospective cohort study	China	2011–2022	Random glucose <140 mg/dL	Random glucose ≥140 mg/dL	2,412	1,856	556
Zhou et al., (2020) [10]	Prospective observational registry study	China	2020	Non-diabetic AMI patients	Diabetic AMI patients	277	182	95
Cui et al., (2021) [11]	Retrospective multicenter observational cohort study	China	2021	Hyperglycemic AMI patients	Non-hyperglycemic AMI patients	1,288	664	624
Cui et al., (2023) [12]	Prospective multicenter registry study (CAMI Registry)	China	2023	AMI patients with diabetes	AMI patients without diabetes	5308	2081	3227
Mamadjanov et al., (2021) [13]	Population-based, observational registry study	Germany	2021	Age 65–74 years	Age 75–84 years	5,530	3,709	1,821
Paolisso et al., (2021) [14]	Multicenter, prospective, observational cohort	Italy	2021	MIOCA	MINOCA	2,431	2,198	233
Upur et al., (2022) [15]	Retrospective, population-based cohort study	China	2023	Non-diabetic AMI patients	Diabetic AMI patients	3,330	2,270	1,060
Ding et al., (2019) [16]	Retrospective observational	China	2018	Euglycemia (≤140 mg/dL)	Moderate hyperglycemia (141–179 mg/dL)	1698	1216	370
Ritsinger et al., (2021) [17]	Nationwide prospective registry cohort	Sweden	2012–2017	Glucose 4.0–6.0 mmol/L (72–109 mg/dL)	Glucose 7.8–11.0 mmol/L (140–198 mg/dL)	45,468	15,581	10,094
Schmitz et al., (2022) [18]	Population-based registry cohort (retrospective analysis)	Germany	2009–2014	Diabetes	No diabetes	2,311	681	1,630

Table 2 demonstrates the variation in the glycemic control, which was defined and measured across the studies included that evaluated the outcome of acute myocardial infarction. Most of the investigations evaluated the glycemic exposure at the time of admission for blood glucose measurements, which indicated the fasting plasma glucose, random glucose, or admission plasma glucose, which reflected the significance of early metabolism. Studies like those by Zhou et al. (2020) [10], Cui et al. (2021) [11], and Schmitz et al. (2022) [18] included the indicators like HbA1c, estimated chronic glucose, and stress hyperglycemia ratios (SHR) for differentiating the acute stress-associated dysglycemia from the chronic diabetes-related hyperglycemia. The duration of assessment was high during the hospital admission or within the initial 24 hours, to ensure the standard risk of evaluation. Table 2 highlighted the methodological heterogeneity but also emphasized glycemia as the crucial indicator for prognosis.

**Table 2:** Glycemic assessment parameters and the hyperglycemia among the included AMI studies AMI: Acute myocardial infarction

Author	Exposure-related parameters	Definition of glycemic control	Glycemic indicators used	Timing of glycemic assessment	Categories/thresholds for good vs poor control
Cui et al., (2022) [7]	Admission hyperglycemia stratified by diabetes status	First fasting plasma glucose at hospital admission	Admission fasting plasma glucose; HbA1c used only to define diabetes (≥6.5%)	At admission (first fasting sample)	Diabetes: ≥14.80 mmol/L (hyperglycemia) vs <14.80 mmol/L. No diabetes: ≥6.77 mmol/L (hyperglycemia) vs <6.77 mmol/L
Ritsinger et al., (2019) [8]	Admission plasma glucose (APG) in AMI patients with and without known diabetes	Glycemic status defined by admission plasma glucose; diabetes defined by reported diagnosis or treatment	Admission plasma glucose only; HbA1c not available; fasting status uncertain	At hospital admission during index AMI	Non-diabetics (WHO criteria): Group I: <6.1 mmol/L; Group II: 6.1–6.9 mmol/L; Group III: 7.0–11.0 mmol/L; Group IV: >11.0 mmol/L. Known diabetes: reported diagnosis
Yuan et al., (2023) [9]	Admission random plasma glucose in AMI patients without diabetes	Glycemic status defined solely by random glucose measured at admission; diabetes excluded by diagnosis/history	Admission random glucose only; HbA1c not measured; fasting glucose not specified; mean glucose not used	At hospital admission during index AMI	Group 1: <140 mg/dL (reference); Group 2: 140–200 mg/dL; Group 3: >200 mg/dL. Risk increase noted ≥122 mg/dL (mortality) and ≥111 mg/dL (cardiogenic shock)
Zhou et al., (2020) [10]	Admission plasma glucose and stress hyperglycemia indices in AMI patients	Glycemic status defined using admission blood glucose (ABG); diabetes defined by history, treatment, or HbA1c ≥6.5%	Admission blood glucose (ABG), HbA1c; estimated chronic blood glucose (CBG); A/C ratio (ABG ÷ estimated CBG)	ABG measured at hospital admission; HbA1c measured during hospital stay	WHO-based glucose categories: Group I: <6.1 mmol/L; Group II: 6.1–6.9 mmol/L; Group III: 7.0–11.0 mmol/L; Group IV: >11.0 mmol/L. Alternative definitions: ABG ≥140 mg/dL or A/C ratio > upper tertile (>1.22)
Cui et al., (2021) [11]	Admission blood glucose (ABG) as prognostic exposure in AMI patients with diabetes, pre-diabetes and normal glucose regulation	Glycemic control defined using admission blood glucose; diabetes status defined by history, treatment, discharge diagnosis, or HbA1c ≥6.5%	Admission blood glucose (ABG); HbA1c measured at admission	ABG measured at hospital admission; HbA1c measured during index hospitalization	ROC-derived ABG cutoffs for two-year mortality: Diabetes: ≥9.0 vs <9.0 mmol/L, Pre-diabetes: ≥7.2 vs <7.2 mmol/L NGR: ≥6.2 vs <6.2 mmol/L
Cui et al., (2023) [12]	Stress hyperglycemia assessed using fasting SHR and fasting plasma glucose (FPG) in AMI patients with and without diabetes	Glycemic control defined by relative stress hyperglycemia (fasting SHR) and absolute fasting glucose levels; diabetes defined by history, hypoglycemic therapy, or HbA1c ≥6.5%	Fasting plasma glucose (FPG), HbA1c; fasting stress hyperglycemia ratio (SHR = FPG ÷ [1.59×HbA1c − 2.59]); admission glucose not primary	First fasting blood sample during hospitalization; HbA1c measured at admission	Quartile-based classification for fasting SHR, FPG, and HbA1c (Q1–Q4); poor control defined as highest quartile (Q4)
Mamadjanov et al., (2021) [13]	Admission blood glucose as exposure for short-term prognosis in older AMI patients	Glycemic control assessed using absolute admission blood glucose; diabetes defined by known status at admission	Admission blood glucose only (first measured glucose); HbA1c not used	First blood glucose measurement at hospital admission	No predefined cut-offs; glucose analysed as continuous variable (per 1 SD increase); age-, diabetes- and MI-type stratified analyses
Paolisso et al.,(2021) [14]	Admission stress hyperglycemia (aHGL) as prognostic exposure in AMI patients with obstructive and non-obstructive coronary arteries	Hyperglycemia defined solely by admission blood glucose ≥140 mg/dL, irrespective of diabetes status	Admission blood glucose only; HbA1c, fasting glucose and mean glucose not used	Single glucose measurement at hospital admission	aHGL ≥140 mg/dL vs no-aHGL <140 mg/dL
Upur et al., (2022) [15]	Admission fasting blood glucose (FBG) as exposure for short- and long-term outcomes in AMI	Glycemic control defined by diabetes-status–specific admission FBG cut-offs	FBG on admission; HbA1c used only to classify diabetes status; admission/random glucose, mean glucose not used	FBG measured within 24 hours of hospital admission	Non-diabetic: <5.6 vs ≥5.6 mmol/L; diabetic: <10.6 vs ≥10.6 mmol/L (derived from restricted cubic spline)
Ding et al.,(2019) [16]	Admission hyperglycemia as a predictor of in-hospital mortality in non-diabetic AMI	Glycemic status defined solely by admission blood glucose categories	Admission glucose (primary exposure); fasting plasma glucose and HbA1c measured but not used for exposure definition; mean glucose not used	First blood glucose test at hospital admission	Euglycemia ≤140 mg/dL; moderate hyperglycemia 141–179 mg/dL; severe hyperglycemia ≥180 mg/dL
Ritsinger et al., (2021) [17]	Admission blood glucose level in patients with AMI without diagnosed diabetes	Glycemic status defined by categorized admission glucose levels in non-diabetic patients	Admission blood glucose (primary exposure); HbA1c analyzed in a subgroup only; fasting state for most patients; mean glucose not used	First blood glucose measurement within 24 hours of hospital admission	Group I: 2.0–3.9 mmol/L; Group II (reference): 4.0–6.0 mmol/L; Group III: 6.1–6.9 mmol/L; Group IV: 7.0–7.7 mmol/L; Group V: 7.8–11.0 mmol/L
Schmitz et al., (2022) [18]	Stress hyperglycemia ratio (SHR) and admission blood glucose in AMI patients with and without diabetes	Stress hyperglycemia quantified by SHR; absolute hyperglycemia assessed by admission glucose	Admission blood glucose; HbA1c measured from stored plasma samples and used to calculate SHR; fasting glucose and mean glucose not used	Admission glucose measured upon hospital admission or during hospital stay; HbA1c measured from blood samples collected during hospitalization	No predefined clinical cut-offs; SHR and admission glucose analyzed as continuous variables using restricted cubic splines

The included studies are used for the analysis of survival rate and the regression-based statistical methods for investigating the association between the glycemic parameters and CV outcomes. Kaplan-Meier analysis with Cox proportional hazards regression was the most crucial strategy, while some of the studies applied logistic regression and comparative statistical tests. The advanced techniques, like restricted cubic splines and ROC curve analysis, were utilized to explore the non-linear relationships and predict the level of accuracy. The estimation suggested that the hazard ratios (HR 1.19-1.44) and the odds ratios (OR 1.48-1.66), and the p-value were maintained at <0.05, which indicated the relationship between hyperglycemia and adverse outcomes. The key founders include age, sex, CV risk factors, clinical severity, and treatment variables. Some of the studies included the subgroup and sensitivity analyses, on the basis of the status of diabetes and clinical features (Table 3).

**Table 3:** Statistical analysis used for the included studies STEMI: ST-elevation myocardial infarction; PCI: percutaneous coronary intervention; MI: myocardial infarction; ACE: angiotensin-converting-enzyme; LDL-C: low-density lipoprotein cholesterol; LVEF: left ventricular ejection fraction; MACE: major adverse cardiovascular event; IPTW: inverse probability of treatment weighting

Author	Statistical and reporting parameters	Effect measures reported	Confidence intervals and p-values	Adjustment variables in multivariate models	Sensitivity or subgroup analyses
Cui et al.,(2022) [7]	Kaplan–Meier survival analysis, log-rank test, Cox regression	Hazard ratios	HR 1.44 (95% CI 1.16–1.79), p = 0.001	Age, sex, prior cardiovascular disease, Killip class, heart rate, CRP, serum creatinine, STEMI, PCI, admission hyperglycemia	Stratified analyses by diabetes status
Ritsinger et al.,(2019) [8]	Event rates by WHO glucose categories; logistic regression analysis	Odds ratios (OR)	HR 1.38 (95% CI 1.11–1.72), p = 0.004	Age, sex, previous myocardial infarction, STEMI vs NSTEMI indication	Stratified by known diabetes vs no diabetes; glucose categories per WHO criteria
Yuan et al., (2023) [9]	Event rates by WHO glucose categories; logistic regression analysis	Odds ratios (OR)	OR 1.52 (95% CI 1.19–1.94), p < 0.001	Age, BMI, SBP, heart rate, ALT, AST, creatinine, hs-CRP, triglycerides, HDL-C, Lp(a), CK, CK-MB, AMI type, revascularisation status; IPW-adjusted models	Stratified by age, sex, hypertension, smoking, alcohol use, SBP, DBP, AMI type, revascularisation status, and eGFR
Zhou et al., (2020) [10]	Chi-square (χ²) test, Kaplan–Meier survival analysis, Student’s t-test, Mann–Whitney U test	Hazard ratio (HR)	OR 1.58 (95% CI 1.21–2.07), p = 0.001	Age, sex, BMI, STEMI, Killip class II–IV, primary PCI, smoking, hypertension, previous MI, previous PCI, heart rate, systolic BP, LVEF, HbA1c, triglyceride, LDL-C, statin at discharge	Subgroups by glucose status (DM, pre-DM, NGR); age, sex, BMI, diagnosis, primary PCI, anterior MI, Killip class, vessel number, revascularization status
Cui et al., (2021) [11]	Kruskal–Wallis H test, Pearson’s Chi-square (χ²) test	Odds ratio (OR)	HR 1.32 (95% CI 1.10–1.58), p = 0.003	Clinically important and statistically significant variables from univariable analysis (demographics, cardiovascular risk factors, clinical parameters, laboratory values, treatments)	Diabetic vs non-diabetic patients; quartiles of fasting SHR, FPG, HbA1c; continuous vs categorical analyses
Cui et al., (2023) [12]	Kaplan–Meier survival analysis, log-rank test, Cox regression	Odds ratio (OR)	HR 1.41 (95% CI 1.09–1.83), p = 0.009	Age, sex, diabetes, reperfusion therapy, ACE inhibitors, beta-blockers, lipid-lowering drugs, antiplatelets, insulin, cardiac arrest, in-hospital complications	Stratified by age (65–74 vs 75–84), diabetes status, STEMI vs NSTEMI; interaction testing
Mamadjanov et al., (2021) [13]	Descriptive statistics, comparative tests, regression modeling with predefined significance threshold,	Odds ratio (OR),	OR 1.48 (95% CI 1.10–1.99), p = 0.01	Demographic characteristics, cardiovascular risk factors, clinical presentation, laboratory parameters, and treatments	Stratified analyses by diabetic status and biomarker categories
Paolisso et al.,(2021) [14]	Descriptive statistics, Chi-square test, Kaplan-Meier survival analysis,	Hazard ratio (HR), Odds ratio (OR) for in-hospital events,	HR 1.36 (95% CI 1.05–1.77), p = 0.02	Age, gender, hypertension, Killip class, AMI phenotype (MIOCA/MINOCA), smoking, LVEF, STEMI/NSTEMI, T2DM, admission hyperglycemia, other clinically relevant covariates,	Stratification by MIOCA vs MINOCA, diabetic vs non-diabetic, hyperglycemic vs normoglycemic, short- and long-term outcome analyses, Cox regression with glucose as continuous variable
Upur et al., (2022) [15]	Survival and clinical events confirmed via records, physician contact, and telephone interviews.	Hazard ratios (HRs), odds ratios (ORs).	SHR AUC 0.6912 (95% CI 0.6317–0.7496); Admission glucose AUC 0.716 (95% CI 0.6572–0.7736), p = 0.0351	Age, gender, Killip class, ethnicity, hypertension, COPD, liver/lung disease, diagnosis (NSTEMI/STEMI), EF, PCI, CABG, ACEI/ARB, beta-blockers.	By age, gender, ethnicity, hypertension, diagnosis, Killip class, EF; additional sensitivity analyses excluding in-hospital deaths, using MACE, without IPTW.
Ding et al.,(2019) [16]	Non-parametric tests for continuous variables, chi-square for categorical; logistic regression for mortality predictors.	Odds ratio (OR).	HR 1.19 (95% CI 1.09–1.31) above 5.6 mmol/L in non-diabetics; HR 1.27 (95% CI 1.10–1.46) above 10.6 mmol/L in diabetics; p < 0.001	Age, log NT-proBNP, PCI, insufficient myocardial reperfusion, admission glucose.	Pairwise comparison of mortality and adverse events between glucose groups using adjusted p-values (α=0.016).
Ritsinger et al., (2021) [17]	Kaplan–Meier curves for cumulative events; Cox proportional hazards regression; restricted cubic splines for continuous glucose analysis	Hazard ratios (HR)	OR 1.006 per 1 mg/dL increase (95% CI 1.004–1.008), p < 0.001; OR 3.51 (95% CI 2.15–5.69) for glucose >200 mg/dL	Age, sex, smoking, creatinine, prior MI/HF/CABG/cancer/dementia/dialysis/hypertension/COPD/renal failure/stroke/PAD, year, hospital, MI type, angiography findings, primary decision after angiography, cardiac shock, discharge medications	HbA1c subgroup (23% of cohort); LVEF <50% vs ≥50% stratification; restricted cubic spline for continuous glucose levels; nonfatal events and subsequent mortality analyzed
Schmitz et al., (2022) [18]	Chi-square/Fisher for categorical, t-test or nonparametric for continuous variables; ROC curves with AUC; Cox regression; restricted cubic splines for nonlinear effects; p<0.05 considered significant	Hazard ratios (HR), Area under curve (AUC)	OR 1.66 (95% CI 1.20–2.31), p = 0.002 for plaque rupture	Age, sex, typical chest pain, smoking, hyperlipidemia, hypertension, impaired renal function, PCI, bypass surgery, lysis therapy, diabetes interaction term	Subgroup by diabetes status (diabetes, prediabetes, no diabetes); ROC curves and mortality analyzed by subgroups; SHR vs admission glucose comparison; non-linear dose-response using restricted cubic splines

Table 4 suggested that dysglycemia was related to the enhanced rate of short- and long-term mortality among AMI patients, specifically among non-diabetic individuals, along with admission hyperglycemia. Several studies have reported the interrelationship between stress hyperglycemia and increased all-cause mortality, while the CV-specific mortality was rarely assessed, and the outcome analysis. Also, some of the studies used stress hyperglycemia indices, which stated improved prognosis of the relative hyperglycemia rather than the absolute glucose levels. Reported all-cause mortality ranges between 9.0% and 16.3%, and the variation of the in-hospital mortality ranges between 1.9% and 9.8% across studies. Short-term mortality ranges from 2.3% to 15.7%, while the prolonged follow-up studies reported a two-year rate of mortality from 11.8% to 15.2%. 

**Table 4:** Effect of glycemic status on mortality of the patients in each included study

Author	All-cause mortality (%)	Cardiovascular mortality (%)	In-hospital mortality (%)	Short-term mortality (%)	Long-term mortality (%)
Ding et al., 2019 [16]	–	–	1.90%	–	–
Cui et al., 2021 [11]	16.30%	–	–	–	16.30%
Cui et al., 2022 [7]	–	–	–	–	11.8–15.2% (two-year mortality across glucose groups)
Cui et al., 2023 [12]	–	–	4.20%	–	–
Ritsinger et al., 2021 [17]	9.00%	–	–	–	9.00%
Mamadjanov et al., 2021 [13]	–	–	–	9.10%	–
Paolisso et al., 2021 [14]	–	57.6%	2.30%	2.30%	–
Ritsinger et al., 2019 [8]	–	–	–	3.7–15.7%	–
Zhou et al., 2020 [10]	–	–	–	–	–
Schmitz et al., 2022 [18]	–	–	–	28-day mortality reported (percentage not specified)	Five-year mortality reported (percentage not specified)
Upur et al., 2022 [15]	25.10%	–	6.60%	6.60%	25.10%
Yuan et al., 2023 [9]	9.80%	–	9.80%	9.80%	–

Table 5 showed negative outcomes apart from the rate of mortality, which were variably defined. Hyperglycemia was related to an increased rate of major adverse cardiac events, including heart failure, cardiogenic shock, and arrhythmic complications. The association with the myocardial infarction or stroke was inconsistent, suggesting the vulnerability of certain pathophysiological pathways. The length of the hospital stay was not impacted by the glycemic status, which indicated that the prognostic impact may not translate into utilization of the prolonged acute care. Thus, the studies with a wide MACE definition stated strong associations, which highlighted that outcome selection is the key determinant of the observed impacts.

**Table 5:** Major adverse cardiac conditions and the cardiovascular outcomes related to dysglycemia among AMI studies AMI: Acute myocardial infarction; MI: myocardial infarction; HF: heart failure

Author	Major adverse cardiac events	Individual MACE components	Length of hospital stay	Readmission rates
Cui et al., (2022) [7]	Composite of all-cause death, non-fatal myocardial infarction, target vessel revascularization, and non-fatal stroke	Non-fatal MI (0.5%), revascularization (4.4%), non-fatal stroke (2.6%)	no significant difference between hyperglycemia and non-hyperglycemia groups	Not reported
Ritsinger et al.,(2019) [8]	Composite of all-cause death, myocardial infarction, stroke, and heart failure hospitalization	Myocardial infarction, stroke, heart failure, hospitalization, and death	Not reported	Heart failure hospitalization included in the outcome
Yuan et al., (2023) [9]	Not defined as a composite MACE	Cardiogenic shock, VT, VF, atrioventricular block, and new stroke	Not reported	Not reported
Zhou et al., (2020) [10]	MACCE defined as all-cause death, recurrent MI, stroke, unplanned revascularization	Recurrent MI, stroke, unplanned revascularization	Not reported	Not reported
Cui et al., (2021) [11]	Not assessed (study evaluated only in-hospital death as the primary endpoint)	Not reported	Not reported	Not reported
Cui et al., (2023) [12]	Combined endpoint of in-hospital complications	Cardiac arrest, recurrent MI, pulmonary oedema, cardiogenic shock, ventricular tachycardia, ventricular bradycardia, ventricular fibrillation	Not reported	Not reported
Mamadjanov et al.,(2021) [13]	Not defined as a composite outcome in the study,	Not included as predefined study outcomes,	Collected clinically but not analyzed or reported as an outcome	Not assessed due to lack of post-discharge follow-up,
Paolisso et al.,(2021) [14]	Composite of reinfarction, stroke, heart failure, arrhythmias, 28.6% in hyperglycemic MIOCA vs 16% no-aHGL, 18.4% in hyperglycemic MINOCA vs 10.3% no-aHGL,	Re-infarction (4.7% vs 4.4%), stroke (0.1–0.2%), heart failure (15.8% vs 10.3% in MIOCA; 15.4% vs 5.2% in MINOCA), urgent revascularization not separately reported,	Median 5–6 days (IQR 4–10),	Re-hospitalization for MI, HF, stroke recorded but exact rates not reported,
Upur et al., (2022) [15]	Higher incidence in hyperglycemia groups; most frequent in hyperglycemia patients with diabetes.	Cardiovascular death, re-hospitalization for AMI, target vessel revascularization (TVR), heart failure, and stroke.	Not reported	Reported indirectly as re-hospitalization for AMI under MACE; no separate readmission rate provided
Ding et al., (2019) [16]	Higher incidence with increasing admission glucose; severe hyperglycemia group highest	Cardiovascular mortality, cardiogenic shock, ischemic stroke, fatal ventricular arrhythmia, and atrioventricular block	No significant difference between groups (median ~8 days); not a key outcome	Not reported
Ritsinger et al., (2021) [17]	Elevated glucose associated with increased risk of MACE	Myocardial infarction (no association), stroke (no association), heart failure (HR 1.40; 95% CI 1.30–1.51), and renal failure	Not reported	Not reported separately; rehospitalizations captured as outcomes for HF, renal failure, MI, and stroke
Schmitz et al., (2022) [18]	Not reported	Not reported; individual events such as MI, stroke, or heart failure were not evaluated as outcome endpoints	Not reported.	Not reported.

Table 6 represented the NOS-based bias asses of the included studies, which demonstrated the high quality of methods. Mostly the studies achieved scores between 7 and 9, which indicated the low overall risk of bias, which is driven by A strong process for selection and adequate comparability between the study groups. Satisfactory outcome assessment was performed, and two studies, those by Ding et al. (2019) [16] and Yuan et al. (2023) [9], showed a weak outcome, leading to moderate risks.

**Table 6:** Summary of bias assessment

Study (Author, Year)	Selection (max 4)	Comparability (max 2)	Outcome (max 3)	Total NOS score (max 9)	Overall risk of bias
Cui et al., 2022 [7]	★★★☆	★★	★★☆	7	Low
Ritsinger et al., 2019 [8]	★★★☆	★★	★★☆	7	Low
Cui et al., 2021 [11]	★★★★	★★	★★☆	8	Low
Cui et al., 2023 [12]	★★★★	★★	★★☆	8	Low
Mamadjanov et al., 2021 [13]	★★★★	★★	★★☆	8	Low
Paolisso et al., 2021 [14]	★★★☆	★★	★★☆	7	Low
Ding et al., 2019 [16]	★★★☆	★★	★☆☆	6	Moderate
Ritsinger et al., 2021 [17]	★★★★	★★	★★★	9	Low
Zhou et al., 2020 [10]	★★★☆	★★	★★☆	7	Low
Schmitz et al., 2022 [18]	★★★★	★★	★★☆	8	Low
Upur et al., 2022 [15]	★★★★	★★	★★☆	8	Low
Yuan et al., 2023 [9]	★★★☆	★★	★☆☆	6	Moderate

Discussion

The incidence and mortality of ACS among diabetic patients are much higher than those among non-diabetics. Hyperglycemia is implicated in vascular damage and cardiac myocyte death through different molecular mechanisms as advanced glycation end products, protein kinase C, polyol pathway flux, and the hexosamine pathway. High free fatty acid (FFA) concentrations may be toxic in acute ischemic myocardium due to several mechanisms, thus leading to endothelial dysfunction. A reduction in free fatty acid plasma levels and high glucose availability can be achieved by using a glucose-insulin-potassium infusion (GIKi) during AMI. DIGAMI studies suggested blood glucose level as a significant and independent mortality predictor, enhancing the significant role of glucose control in their management [19]. Patients who underwent type 2 diabetes along with percutaneous coronary intervention described the relationship between poor glycemic control and the enhanced prolonged mortality. Higher baseline HbA1c levels (>10%) were associated with a higher mortality risk compared to well-controlled patients (HbA1c ≤7%). Type 2 diabetes patients underwent PCI, with poor glycemic control and high baseline HbA1c levels (>10%), which was related to the rise of CV mortality and adverse cardiac events. This suggested that chronic hyperglycemia is an independent predictor of unfavorable CV outcomes. The incidence and mortality of ACS were significantly higher among diabetic patients than in non-diabetics. Hyperglycemia contributed to vascular injury and cardiomyocyte death through various mechanisms, such as advanced glycation end products, protein kinase C activation, oxidative stress, endothelial dysfunction, inflammation, and altered polyol and hexosamine pathways. These processes help to promote the activation of platelets, impaired nitric oxide availability, microvascular dysfunction, and accelerated atherosclerosis. The high fatty acids at the time of ischemia cause impaired myocardial metabolism and enhance cardiac injury. GIKi reduces the circulation of FFAs for better usage of the myocardial glucose. A high level of HbA1c worsens the metabolic pattern and may present acutely, as evidenced by an elevated ST segment. The association was noted among non-insulin users, whereas the insulin-treated patients showed no rate of mortality. The results suggested that chronic hyperglycemia is a significant independent predictor of adverse CV outcomes [20]. A high level of admission plasma glucose is associated with increased mortality among patients with ACS. The tight glycemic control by the use of insulin infusion among acute coronary syndrome patients has improved the clinical outcome. Patients with consistent insulin infusion received improved glycemic control rather than subcutaneous insulin. The infusion showed low rates of heart failure cases for 7 and 30 days, with a reduction in the hemodynamic instability during ICU stay. Significantly, the rate of mortality was low in the case of the intensive control group, without any deaths reported, rather than high mortality cases in the conventional group. This revealed the significant impact of strict glycemic management on reducing short-term complications and improving the prognosis [21]. The rise of diabetes condition during both 30-day and one-year mortality after ACS, with enhanced risk for STEMI and UA/NSTEMI patients, highlighted the poor prognosis of the CV conditions [22].

## Conclusions

The review concluded that the glycemic status is a strong and consistent predictor for analyzing the adverse outcome among T2DM patients presenting with ACS. Across the robust observational evidence, admission hyperglycemia and chronic glycemic markers such as HbA1c were related to the enhanced risk of in-hospital, short-term, and long-term mortality. Significantly, hyperglycemia is a strong predictor for high incidence of major cardiac events, specifically, heart failure, cardiogenic shock, and arrhythmias, although an inconsistent relation was observed for myocardial infarction and stroke. The findings indicated that acute dysglycemia, such as stress hyperglycemia, is a strong predictor for the early outcome rather than the pre-existing diabetes status. The direction of the association was the same across all of the included studies. However, the benefits of intensive glycemic control were inconclusive, with the potential risks of hypoglycemia. The review paper showed the significance of the glycemic evaluation and the individualized management strategies among ACS patients with diabetes, while triggering the requirement for more high-quality randomized trials to establish the optimal level of therapeutic targets. The future perspective suggests that while standard glycemic targets provide a general framework, glycemic control should be individualized based on patient characteristics, with appropriate timing of intervention and safe therapeutic approaches to balance glucose levels and minimize hypoglycemia-related complications, particularly among high-risk cardiovascular populations.
